# Proactive inhibition deficits with normal perfusion after pediatric mild traumatic brain injury

**DOI:** 10.1002/hbm.24778

**Published:** 2019-08-28

**Authors:** Andrew R. Mayer, David D. Stephenson, Christopher J. Wertz, Andrew B. Dodd, Nicholas A. Shaff, Josef M. Ling, Grace Park, Scott J. Oglesbee, Ben C. Wasserott, Timothy B. Meier, Katie Witkiewitz, Richard A. Campbell, Ronald A. Yeo, John P. Phillips, Davin K. Quinn, Amy Pottenger

**Affiliations:** ^1^ The Mind Research Network/LBERI Albuquerque New Mexico; ^2^ Department of Psychology University of New Mexico Albuquerque New Mexico; ^3^ Department of Neurology University of New Mexico Albuquerque New Mexico; ^4^ Department of Psychiatry University of New Mexico Albuquerque New Mexico; ^5^ Emergency Medicine University of New Mexico Albuquerque New Mexico; ^6^ Department of Neurosurgery Medical College of Wisconsin Milwaukee Wisconsin; ^7^ Department of Cell Biology Neurobiology and Anatomy, Medical College of Wisconsin Milwaukee Wisconsin

**Keywords:** blood oxygen‐level dependent response, cerebral blood flow, pediatric mild traumatic brain injury, response inhibition

## Abstract

Although much attention has been generated in popular media regarding the deleterious effects of pediatric mild traumatic brain injury (pmTBI), a paucity of empirical evidence exists regarding the natural course of biological recovery. Fifty pmTBI patients (12–18 years old) were consecutively recruited from Emergency Departments and seen approximately 1 week and 4 months post‐injury in this prospective cohort study. Data from 53 sex‐ and age‐matched healthy controls (HC) were also collected. Functional magnetic resonance imaging was obtained during proactive response inhibition and at rest, in conjunction with independent measures of resting cerebral blood flow. High temporal resolution imaging enabled separate modeling of neural responses for preparation and execution of proactive response inhibition. A priori predictions of failed inhibitory responses (i.e., hyperactivation) were observed in motor circuitry (pmTBI>HC) and sensory areas sub‐acutely and at 4 months post‐injury. Paradoxically, pmTBI demonstrated hypoactivation (HC>pmTBI) during target processing, along with decreased activation within prefrontal cognitive control areas. Functional connectivity within motor circuitry at rest suggested that deficits were limited to engagement during the inhibitory task, whereas normal resting cerebral perfusion ruled out deficits in basal perfusion. In conclusion, current results suggest blood oxygen‐level dependent deficits during inhibitory control may exceed commonly held beliefs about physiological recovery following pmTBI, potentially lasting up to 4 months post‐injury.

## 1 INTRODUCTION

Pediatric mild traumatic brain injury (pmTBI) represents a significant public health concern due to the sheer volume of new cases (~750,000) that occur each year (Lincoln et al., 2011; Zemek et al., 2017). Current public concerns about concussions (herein used synonymously with pmTBI) are primarily driven by histopathological results obtained at autopsy in select samples of professional athletes (McKee et al., 2013). Conversely, the etiology of post‐concussive symptoms (PCS), the natural course of biological recovery and the potential for long‐term neurological changes are all largely unknown in children (Rausa, Anderson, Babl, & Takagi, 2018). Preclinical and emerging clinical data from adult studies suggest multiple white and gray matter abnormalities may exist post‐injury, with each pathology exhibiting unique time‐courses for recovery (Barkhoudarian, Hovda, & Giza, 2016; Mayer, Mannell, Ling, Gasparovic, & Yeo, 2011; Meier et al., 2015).

Neurovascular coupling has been shown to be disrupted in animal models of trauma (Jang et al., 2017), and the blood oxygen‐level dependent (BOLD) response is frequently used as a proxy measure of coupling in humans (Hillman, 2014). BOLD activity can be quantified both during rest and during cognitive tasks, when patients are more likely to complain of increased symptom burden (Mayer et al., 2018). Specifically, immediately following injury patients frequently report increased symptoms during cognitive testing (Gioia, Schneider, Vaughan, & Isquith, 2009), and objective cognitive deficits are most frequently observed in the domains of attention, processing speed and inhibition (Li & Liu, 2013). In the cognitive neuroscience literature, deficits in response inhibition are measured in response to either reactive (i.e., inhibiting a prepotent motor responses after the stimulus occurs) or proactive (i.e., inhibiting responses in a planned and purposeful manner) demands (Criaud & Boulinguez, 2013). Proactive inhibition typically results in a negative or suppressed BOLD response in healthy motor circuitry in contrast to hyperactivation in psychopathology (Li, Lai, & Chen, 2013; Mayer et al., 2016).

To date, only a handful of studies have examined BOLD deficits using functional magnetic resonance imaging (fMRI) in well‐defined, sub‐acute pmTBI samples (Mayer, Kaushal, et al., 2018), with a single prospective pmTBI study (Hammeke et al., 2013). Posterior cerebellar hyperactivation (pmTBI > healthy controls [HC]) has been observed during inhibitory control (Krivitzky et al., 2011), which also correlated with PCS. Other studies reported hypoactivation (HC > pmTBI) within deep gray matter areas during auditory orienting (Yang et al., 2012) or within frontoparietal regions during working memory (Keightley et al., 2014). Finally, working memory deficits and hypoactivation (pmTBI < HC) of the right frontoparietal and occipital regions were observed 1 day post‐injury, with a reversal (pmTBI > HC) at 13 weeks (Hammeke et al., 2013).

Independent measures of cerebral blood flow (CBF) can disambiguate resting perfusion from other BOLD deficits following trauma (Mayer, Kaushal, et al., 2018). Hypoperfusion in the medial–temporal regions has been observed within 72 hr of injury and again at 3 months following pmTBI (Agrawal, Gowda, Bal, Pant, & Mahapatra, 2005), with deficits also associated with prolonged PCS. Findings of decreased CBF were replicated in the acute phase of sports‐related concussion (Maugans, Farley, Altaye, Leach, & Cecil, 2012), persisting up to 2 months post‐injury. In contrast, other studies report increased CBF at 2 and 6 weeks post‐injury following pmTBI (Stephens, Liu, Lu, & Suskauer, 2018), or increased global CBF in symptomatic pmTBI patients and lower global CBF in asymptomatic patients (Barlow et al., 2017). To the best of our knowledge, no studies have examined BOLD and CBF abnormalities prospectively in the same sample of pmTBI.

The current study used high temporal resolution sampling in conjunction with deconvolution to disambiguate BOLD response during the planning and execution phases of proactive response inhibition. Independent measures of cerebral perfusion and resting state MRI were also collected. Based on previous studies in HC and other patient samples (Li et al., 2013; Mayer et al., 2016), we hypothesized that pmTBI patients would exhibit proactive response inhibition deficits (i.e., hyperactivation) in motor circuitry in conjunction with hypoperfusion during the sub‐acute injury phase (within weeks of injury), with both measures recovering at 4 months post‐injury (early chronic visit). We also predicted that these inhibitory motor deficits would be associated with reduced functional connectivity in motor circuitry, as well as being associated with persistent PCS.

## 2 MATERIALS AND METHODS

### 2.1 Participants

Fifty pmTBI patients (12–18 years old) were consecutively recruited from local Emergency Department and Urgent Care settings in this prospective cohort design. Inclusion criteria were based on the American Congress of Rehabilitation Medicine and the Zurich Concussion in Sport Group (see Supporting Information). Specifically, all pmTBI experienced a closed head injury with Glasgow Coma Score ≥13 when evaluated at the emergency room, an alteration in mental status or at least two new symptoms, a loss of consciousness less than 30 min, and post‐traumatic amnesia limited to 24 hr. Age‐ and sex‐matched HC (*N* = 53) were recruited and scanned at equivalent time points to control for neurodevelopment and/or repeat assessment. The first visit for pmTBI occurred 6.9 ± 2.4 days post‐injury (maximum = 11 days), with the second visit occurring approximately 4 months later (120.4 ± 11.6 between visits). HC were recruited from the local community through word of mouth or fliers and were examined at similar intervals (119.4 ± 12.1 days between visits). Supporting Information and Table S1 detail sample size and demographic information for each imaging analysis, with final sample sizes also presented for each analyses.

Exclusion criteria included history of (a) neurological diagnosis, (b) previous head injury with greater than 30 min loss of consciousness, (c) developmental disorder (autism spectrum disorder or intellectual disability), (d) history of any psychiatric disorders other than adjustment disorder, (e) contraindications for MRI including pregnancy, (f) non‐English speaking or (g) history of substance abuse/dependence for both groups. pmTBI were excluded for any current procedure requiring general anesthesia. HC were also excluded if diagnosed with Attention deficit hyperactivity disorder or a learning disability. Urine‐based drug screens (see Supporting Information) were conducted for all participants at both visits, with only a single pmTBI patient testing positive for medical marijuana. In addition, one pmTBI participant was taking medication that could have potentially affected brain functioning. Participants provided informed consent according to University of New Mexico School of Medicine guidelines.

### 2.2 Clinical and behavioral measures

A Common Data Element focused battery of clinical and neuropsychological measures was administered (see Supplemental Materials/Table 1 for primary vs. secondary measures). Retrospective ratings (i.e., estimation for 1 month ago) of symptoms (Table S2) were also acquired at Visit 1. Participants completed the Post‐Concussion Symptom Inventory (self and parental report), Patient Reported Outcomes Measurement Information System (anxiety, depression, and sleep scales), a self‐report pain rating (0–10 Likert scale), Headache Impact Test, the Strengths and Difficulties Questionnaire, Conflict and Behavioral Questionnaire and self‐reported Tanner stage of development. Outcomes were assessed with the Pediatric Quality of Life Inventory (Generic Core) and Glasgow Outcome Scale Extended (Pediatric Revision). Parental report of psychopathology was measured with the Brief Symptom Inventory.

**Table 1:** Clinical and neuropsychological data

	Outcome	SA HC	SA pmTBI	EC HC	EC pmTBI
Demographics					
Age	D	15.31 ± 1.99	15.73 ± 2.14	–	–
Sex (% male)	D	62.0%	59.5%	–	–
Previous Hx of mTBI*	D	10.0%	26.2%	–	–
Symptom measures					
PCSI«	P	2(0–4.75)	12(4–36.5)	3.5(0.25–8.5)	7.5(0.25–21.25)
PCSI (parent)»«	P	1(0–3)	12(5–29.5)	1.5(0–6)	5.5(0–12.25)
PROMIS sleep*	S	13.58 ± 4.82	18.76 ± 6.23	14.40 ± 4.77	18.86 ± 7.66
PROMIS anxiety«	S	1(0–2)	2.5(0–8.75)	1(0–4)	2(0–6)
PROMIS depression*	S	0(0–3)	1(0–7.75)	1(0–3)	1(0–4)
Pain scale«	S	0(0–1)	3(2–6)	0(0–1)	0(0–2)
HIT‐6«	S	38(36–44.75)	50(46.25–57.75)	40(38–45.5)	48(42–56.75)
BSI (parent)	S	0(0–3)	3(1–5.5)	1.5(0–5.25)	2(0–6)
Behavioral measures					
CBQ»	P	1(0–2)	1(0–2)	0.5(0–2)	1(0.25–3.75)
CBQ (parent)*	P	1(0–3)	1(0.5–4)	0(0–2)	1(0.25–5)
SDQ (parent)	S	–	–	4(2–8.25)	6(3.25–10)
Outcome measures					
PedsQL	P	–	–	88.53 ± 10.11	84.78 ± 9.70
PedsQL (parent)*	P	–	–	87.75 ± 11.37	77.35 ± 14.97
GOS‐E (parent)	S	1(1–1)	2(1–4)	1(1–1)	1(1–1)
Cognitive measures					
TOMMe10	S	9.78 ± 0.51	9.74 ± 0.59	9.78 ± 0.46	9.64 ± 0.82
WRAT‐IV	S	56.08 ± 10.85	52.02 ± 10.70	56.21 ± 9.89	51.46 ± 10.77
PS	P	49.43 ± 6.87	48.06 ± 6.59	51.54 ± 7.76	52.10 ± 8.32
AT	P	48.87 ± 7.33	48.36 ± 7.61	49.89 ± 7.00	49.49 ± 7.50
WM	S	49.93 ± 10.09	46.91 ± 7.20	50.33 ± 9.62	48.73 ± 8.84
EF	S	48.57 ± 6.41	46.95 ± 6.60	51.95 ± 5.65	49.94 ± 7.39
Computerized testing					
PS	P	2.51 ± 0.06	2.53 ± 0.07	2.50 ± 0.06	2.52 ± 0.08
AT*	P	2.69 ± 0.04	2.72 ± 0.07	2.69 ± 0.05	2.71 ± 0.06
WM	S	2.88 ± 0.08	2.87 ± 0.08	2.85 ± 0.07	2.86 ± 0.11
Mem	S	2.99 ± 0.07	3.00 ± 0.09	2.99 ± 0.07	2.98 ± 0.09

Abbreviations: AT, attention; BSI, brief symptom inventory; CBQ, Children's Behavior Questionnaire; D, demographic; EC, early chronic; EF, executive functioning; GOS‐E, Extended Glasgow Outcome Scale; HC, healthy control; HIT‐6, Headache Impact Test; Hx, history; Mem, memory; P, primary; PCSI, Post‐Concussion Symptom Inventory; PedsQL, Pediatric Quality of Life Inventory; pmTBI, pediatric mild traumatic brain injury; PROMIS, Patient‐Reported Outcomes Measurement Information System; PS, processing speed; S, secondary; SA, sub‐acute; SD, standard deviation; SDQ, Strengths and Difficulties Questionnaire; TOMMe10, Test of Memory Malingering‐Errors on First Ten Items; WM, working memory; WRAT‐IV, Wide Range Achievement Test IV. Data are either formatted at mean ± standard deviation or median (interquartile range). The following symbols denote significant effects: *, main effect of Group; «, Group × Visit interaction with group level differences at sub‐acute visit; », Group × Visit interaction with group level differences at early chronic visit; »«, significant Group × Visit interaction with differences at both visits.

Estimates of premorbid cognitive abilities and effort were obtained along with computerized neuropsychological tests, selected subtests from the Delis–Kaplan Executive Function System and age appropriate versions of Wechsler scales (see Supporting Information). Raw scores were age‐corrected and aggregated into composites for domains of attention, processing speed, working memory, and executive function (primary outcomes).

### 2.3 Task paradigms

**Figure 1:**
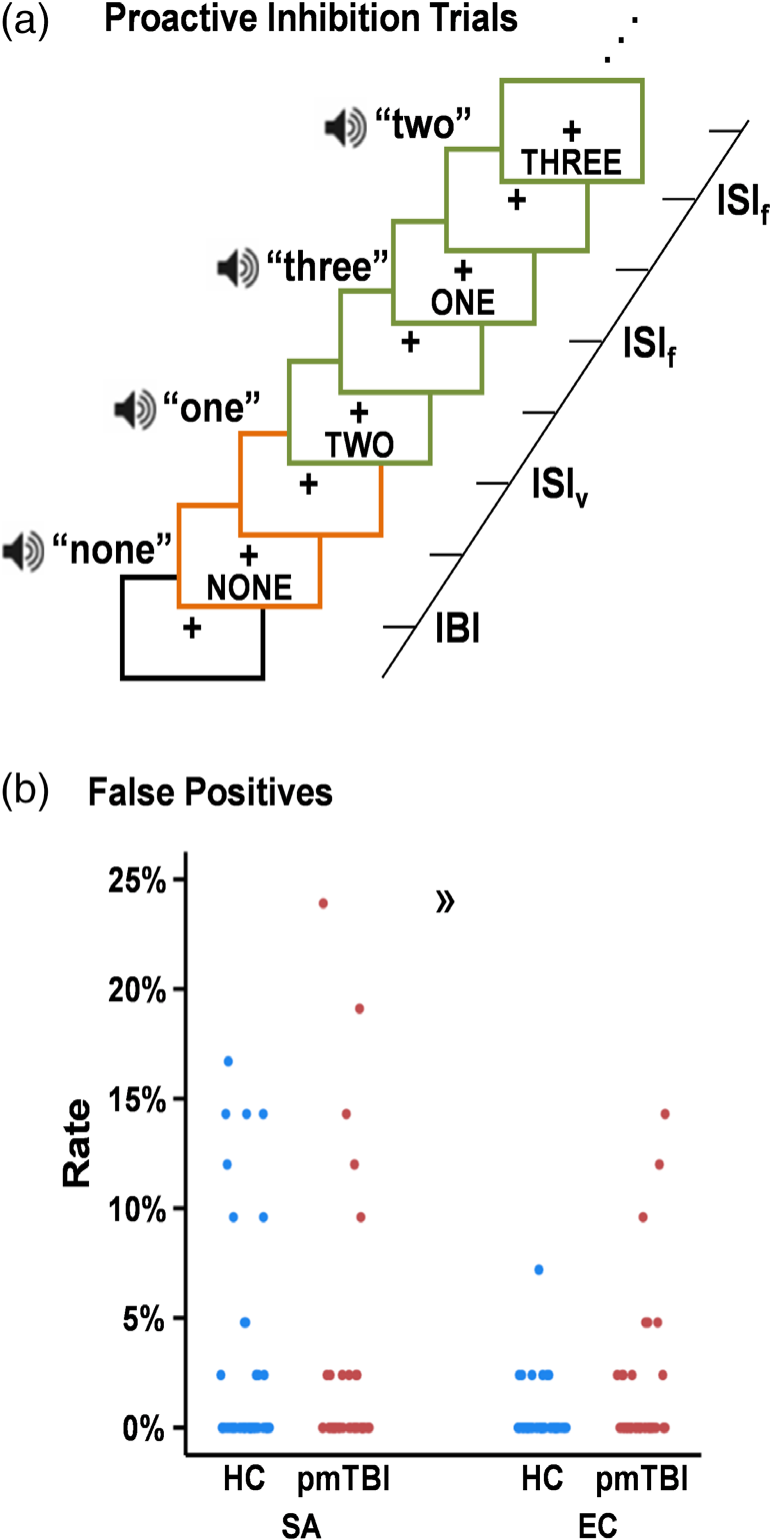
Proactive response inhibition task. A cartoon representation of proactive response inhibition trials (a). Each trial was separated into distinct cue (the word “NONE”; orange colored boxes) and target (written Arabic numerals between one and three; green colored boxes) phases with corresponding HRFs. This was accomplished by having variable inter‐stimulus intervals (ISI_v_) between the cue and the first numeric target as well as variable inter‐block intervals (IBI; black colored box). The inter‐stimulus interval between numeric targets within each block was fixed (ISI_f_). Scatterplots (b) depict the percentage of false‐positive responses for both healthy controls (HC; blue) and pediatric mild traumatic brain injury patients (pmTBI; red) at both the sub‐acute (SA) and early chronic (EC) visits. The directional guillemet (») denotes increased false positive responses for pmTBI at the EC visit, predominantly driven by five participants [Color figure can be viewed at http://wileyonlinelibrary.com]

A full description of the proactive response inhibition task has previously been reported (Mayer et al., 2016). Briefly, a multisensory cue (audiovisual; 300 ms duration) was presented at the beginning of each block (Figure 1a), determining the modality for focused attention (“HEAR” = attend‐auditory; “LOOK” = attend‐visual) or for the planned inhibition of upcoming motor responses (“NONE” = proactive response inhibition). Multisensory numeric targets (words = “ONE,” “TWO,” or “THREE”; 300 ms duration) were presented 2,460–3,380 ms following the cue, with targets occurring at a frequency of 0.66 Hz (6 trials per each 8 s block). Targets were congruent (i.e., same auditory/visual number) or incongruent (i.e., different auditory/visual number) in nature. In the attend‐auditory and attend‐visual conditions, participants responded via a right‐handed button press to one of three target buttons corresponding to the target stimulus in the attended modality while ignoring simultaneously presented numbers in the opposite sensory modality. Participants were instructed to withhold a motor response following cues (“NONE”) as a measure of proactive inhibition. Inhibition should theoretically be greatest after the presentation of the multisensory cue. As such, targets were always incongruent on NONE trials. All stimuli were presented foveally and binaurally via headphones (head‐centered).

Inter‐block intervals were jittered (3,700–5,540 ms) to minimize nonlinear summing of the hemodynamic response function (HRF; Glover, 1999) and decrease temporal expectations between the cue and the target. The task design resulted in a nonsingular/invertible matrix with only moderate collinearity. Active trial data (attend‐auditory and attend‐visual) collapsed across both groups empirically defined motor circuitry for a priori analyses. For the resting state paradigm, participants attended to a foveally presented fixation cross for 5 min and 6 s.

### 2.4 MR imaging parameters

MRI data was acquired on a 3T Siemens Trim Trio scanner with a 32‐channel head coil (see Supporting Information). High resolution T_1_‐weighted (1 × 1 × 1 mm), T_2_‐weighted (1.1 × 1.1 × 1.5 mm), susceptibility weighted images (1.0 × 1.0 × 1.5 mm) and fluid attenuated inversion recovery images (0.8 × 0.8 × 3.0 mm) were collected and reviewed by a blinded, board certified radiologist.

Task (2 runs) and connectivity (1 run) data were acquired utilizing a single‐shot, gradient‐echo echoplanar pulse sequence with 56 interleaved slices acquired for whole‐brain coverage (3.02 × 3.02 × 3.00 mm) using multiband imaging to achieve high temporal sampling (TR = 460 ms) of the HRF. The initial images from task (1,208 final images) and resting (647 final images) state runs were excluded to account for T_1_ equilibrium effects. A reference image (multiband factor = 1) was also acquired to facilitate registration to T_1_‐weighted data due to increased gray‐white contrast. Two spin‐echo field mapping sequences (3.02 × 3.02 × 3.00 mm) with reversed phase encoding directions (A ➔ P; P ➔ A) were generated to correct for susceptibility related distortion.

Pseudo‐Continuous Arterial Spin Labeling (pCASL; 45 tagged/untagged images) and proton density sequences were acquired with 20 interleaved slices for whole brain coverage of resting state perfusion (3.44 × 3.44 × 6.00 mm). The post‐labeling delay (1,800 ms) was determined both on recommended values for adolescents (Alsop et al., 2015) and predictions of trauma‐related hypoperfusion (Barkhoudarian et al., 2016; Meier et al., 2015).

### 2.5 MR processing and analysis

Task and resting state data were first assessed for anomalous values and replaced using AFNI's despiking tool (Cox, 1996). Second, images were temporally interpolated to the first slice, then spatially registered to a reference image in two‐ and three‐dimensional space using AFNI software suite tools. Fourth, images were corrected for susceptibility distortions using FSL's Topup algorithm (Andersson, Skare, & Ashburner, 2003; Smith et al., 2004). Fifth, images were converted to standard stereotaxic space (Talairach & Tournoux, 1988) using a nonlinear transformation (AFNI 3dQwarp) and then finally spatially blurred (6‐mm Gaussian filter).

A single HRF was generated using finite impulse response deconvolution for both the cue (14.26 s post‐cue onset) and target (20.70 s post‐target onset) phases of the task on our high temporal resolution data. Nuisance regressors modeled variance associated with 12 motion parameters, error trials as well as a second‐order polynomial to reduce hardware‐related effects (Mayer et al., 2011, 2019). Beta coefficients were then separately summed across individual HRF periods to obtain estimates of peak (2.76–6.44 s) and inhibitory (6.90–11.04 s) activity during the cue phase. Similarly, beta coefficients were also summed across the target HRF to obtain estimates of peak (2.30–5.98 s) and late peak (5.98–9.66 s) activity. Summed beta values were then divided by the average model intercept to estimate percent signal change (PSC). Resting state functional connectivity maps were calculated by regressing motion parameters and their derivatives, as well as estimates of physiological noise from white matter and cerebral spinal fluid, followed by the application of a bandpass filter (0.01–0.1 Hz) to the data.

pCASL images were despiked and registered to the PD image using two‐ and three‐dimensional motion correction algorithms from the AFNI software suite. Both images were then spatially blurred using a 6‐mm Gaussian kernel. Each preprocessed labeled image was next subtracted from the paired control image, after which CBF was quantified using in‐house software based on established parameters [blood/tissue water partition coefficient = 0.9 ml/g; longitudinal relaxation time of blood = 1,664 ms; labeling efficiency = 0.85; label duration = 1,665 ms] and algorithms (Wang et al., 2005). T_1_ magnetization correction and scaling of CBF was accomplished on a voxel‐wise basis with the PD image. The quantified CBF data were then averaged and spatially transformed to stereotaxic space in a manner identical to fMRI data.

### 2.6 Statistical analyses

pmTBI and HC were compared on key demographics to ensure effective matching using parametric or nonparametric tests as appropriate. For both primary and secondary (see Table 1) clinical measures, data were modeled with either negative binomial, gamma or normal distributions using 2 × 2 [Group (pmTBI vs. HC) × Visit (Sub‐acute vs. Early chronic)] generalized estimating equations as appropriate. Retrospective ratings were used as a covariate when available. Simple effects testing of significant interactions were conducted with generalized linear models, and incidence rate ratios (IRR) are reported. Multiple regression investigated relationships between imaging and primary outcome measures.

Based on previous publications (Mayer et al., 2016), a priori regions of interest included left premotor cortex, bilateral supplemental motor area (SMA), and left sensorimotor cortex (SMC), all of which were empirically defined based on evoked motor responses (i.e., button presses) during the attend‐auditory and attend‐visual conditions (Supporting Information and Figure S2). For each ROI, separate 2 × 2 [Group (pmTBI vs. HC) × Visit (Sub‐acute vs. Early chronic)] ANCOVAs were respectively performed examining both peak and inhibitory activity during the cue HRF and CBF data using mean framewise displacement (FD) as a covariate.

In addition, 2 × 2 [Group (pmTBI vs. HC) × Visit (Sub‐acute vs. Early chronic)] whole‐brain or 2 × 2 × 2 [Group (pmTBI vs. HC) × Visit (Sub‐acute vs. Early chronic) × Phase (Peak activation vs. Late peak)] ANCOVAs respectively examined for any other regions exhibiting BOLD abnormalities during the cue or target phase. Finally, 2 × 2 [Group (pmTBI vs. HC) × Visit (Sub‐acute vs. Early chronic)] whole‐brain ANCOVAs were performed to test a priori predictions of CBF hypoperfusion and decreased connectivity.

Whole‐brain results within each modality were corrected for family‐wise error using statistical (*p* < .001) and minimum volume thresholds based on 10,000 Monte Carlo simulations and spherical autocorrelation estimates (Cox, Chen, Glen, Reynolds, & Taylor, 2017). Volume thresholding varied across imaging modalities based on smoothness estimates (fMRI = 574 μl; fcMRI = 848 μl; CBF = 568 μl). Primary imaging figures combine data for any main effects whereas supplemental figures separately plot data for sub‐acute and early chronic visits to eliminate concerns about power for interactions in functional data.

## 3 RESULTS

### 3.1 Clinical, neuropsychological, and behavioral analyses

The pmTBI and HC groups did not differ in terms of handedness, age, self‐reported Tanner stage of development or biological sex (all *p*'s > .10). Significant group differences were observed for previous history of head injuries (*Χ* ^2^ = 4.17, *p* = .041) with pmTBI (26.2%) reporting a greater prevalence relative to HC (10.0%). GLM models indicated that pmTBI reported more symptoms than HC on retrospective ratings of sleep, pain, headache, and self‐reported behavioral problems in conjunction with lower quality of life (all *p*'*s* < .05; Table S2). Groups were statistically similar on retrospective PCS ratings (child and parent), anxiety, depression, parental reports of behavior, and parental quality of life ratings (all *p*'*s* > .05). Groups were also similar for measures of premorbid reading ability and effort (all *p*'*s* > .05). There were no significant effects or interactions for parent reported self‐psychopathology (all *p*'s > .05).

**Figure 2:**
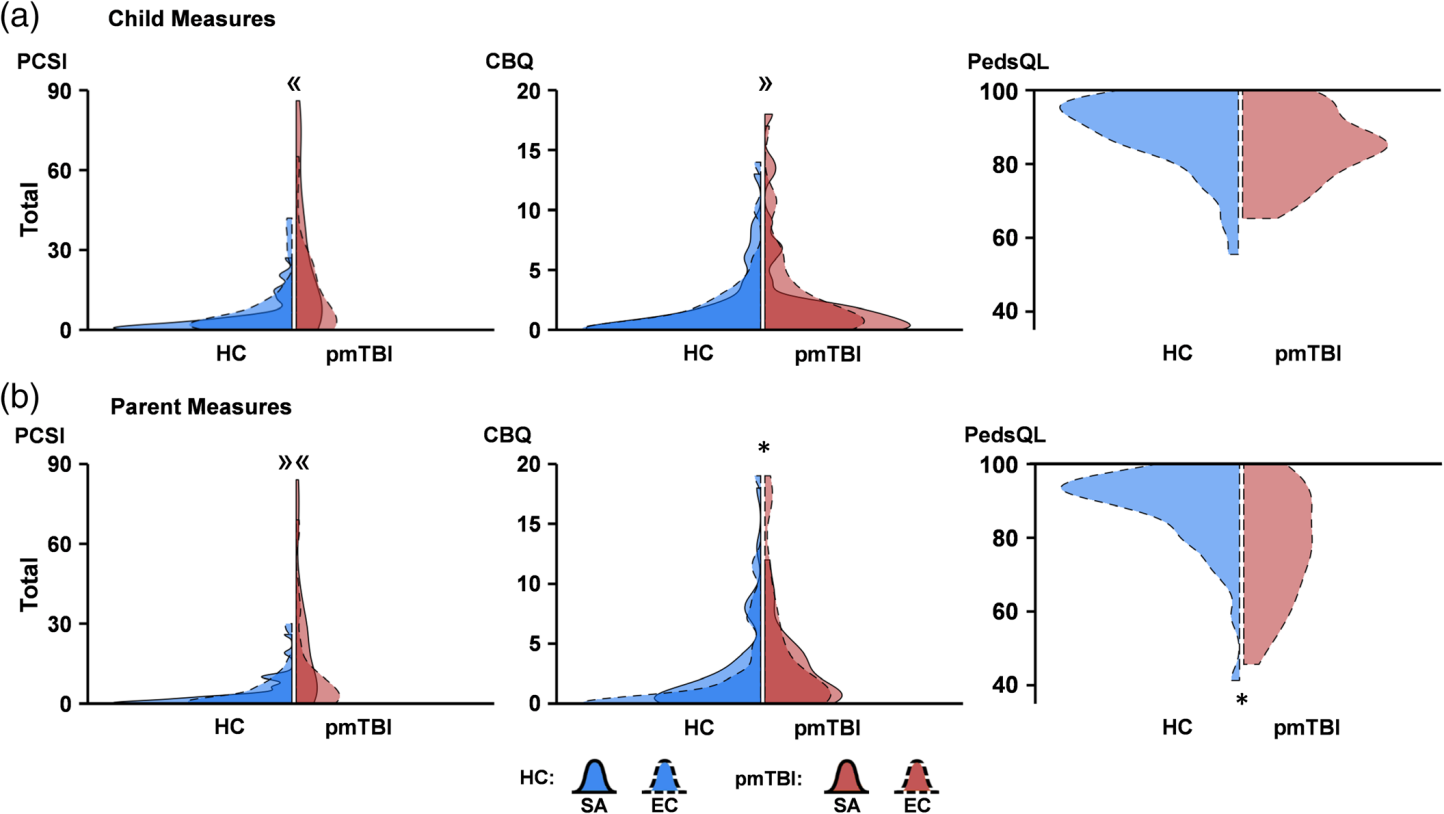
Violin plot distribution of clinical measures. Violin plots showing distributions of primary clinical measures for healthy controls (HC; blue shading) and pediatric mild traumatic brain injury patients (pmTBI; red shading) at sub‐acute (SA; solid line) and early chronic (EC; dashed line) visits. The darker color denotes where the SA and EC distributions overlapped, whereas the lighter color denotes where the SA (light color with solid line) or EC (light color with dashed line) distribution was unique. Data are plotted relative to the densest point among the four distributions in each panel. Self‐ (a) and parent‐report (b) measures include the Post‐Concussion Symptom Inventory (PCSI), Children's Behavioral Questionnaire (CBQ), and Pediatric Quality of Life Inventory (PedsQL; EC only). Significant main effects of group are denoted with an asterisk (*), while significant interactions are denoted by directional guillemets (« = group differences at SA only; » = group differences at EC only; »« = differences in magnitude across SA and EC visits) [Color figure can be viewed at http://wileyonlinelibrary.com]

The analysis for self‐report PCS indicated a significant Group × Time interaction (Wald‐χ^2^ = 14.90; *p* < .001; Table 1; Figure 2a). Simple effects testing demonstrated increased PCS at the sub‐acute phase (pmTBI > HC; IRR = 6.30) but not early chronic phase. The Group × Time interaction for parental PCS ratings was also significant (Wald‐χ^2^ = 7.01; *p* = .008; Figure 2b), but simple effects testing indicated significantly increased symptoms (pmTBI > HC) at both sub‐acute (IRR = 7.69; *p* < .001) and early chronic (IRR = 2.94; *p* = .001) time‐points. Similar to child PCS ratings, secondary measures of anxiety, headaches, and pain (all *p*'*s* < .010; see results included in Supporting Information) indicated significant group differences (pmTBI > HC) at sub‐acute but not early chronic phases. In contrast, only main effects of Group were observed for depression (*p* = .011) and sleep disturbances (*p* = .007), suggestive of prolonged symptomatology up to 4 months post‐injury.

The analysis for self‐report ratings of child behavior demonstrated a significant Group × Time interaction (Wald‐χ^2^ = 6.79; *p* = .009; Figure 2a), with simple effects testing indicating nonsignificant differences for more pmTBI behavioral issues at the early chronic (Wald‐χ^2^ = 3.44; *p* = .064; IRR = 1.54) but not sub‐acute time‐point. The primary parental report of child behavior ratings was significant for the main effect of Group (Wald‐χ^2^ = 4.16; *p* = .041; Figure 2b), with pmTBI parents (IRR = 1.59) reporting elevated values compared to HC parents. A secondary parental measure of behavioral issues/family dynamics was not significant (*p* > .05). Although self‐reported quality of life was not significantly different across groups (*p* > .05; Figure 2a), a main effect was observed for parental ratings (Wald‐χ^2^ = 6.53; *p* = .011; IRR = 0.92; Figure 2b), with pmTBI parents reporting lower quality of life.

Pen and paper measures of attention were not significant (*p*'*s* > .05), with a nonsignificant Group × Time interaction (Wald‐χ^2^ = 3.31; *p* = .069; Figure S1) observed for processing speed. Secondary pen and paper domains of working memory and executive functioning were not significant (all *p*'*s* > .05). There were significant (Wald‐χ^2^ = 5.05; *p* = .025; IRR = 1.03) and nonsignificant (Wald‐χ^2^ = 3.76; *p* = .052; IRR = 1.03) main effects of Group for computerized tests of attention and processing speed, respectively, with pmTBI exhibiting increased reaction times relative to HC. Secondary computerized measures of working memory and learning were not significant (all *p*'*s* > .05).

### 3.2 Behavioral task results

Behavioral results indicated a significant Group × Time interaction (Wald‐χ^2^ = 4.17; *p* = .041), with pmTBI exhibiting a higher number of false positives (*p* = .020; IRR = 3.44) relative to HC at the early chronic but not sub‐acute phase (Figure 1b). An examination of Figure 1b suggests that the difference at the early chronic stage was primarily driven by performance from five pmTBI (findings no longer significant when these individuals were eliminated from analysis). Moreover, the majority of individuals had one or fewer errors during both the sub‐acute (HC: 41/50, 82%; pmTBI: 37/42, 88%) and early chronic (HC: 49/50, 98%; pmTBI: 36/42, 86%) phases, suggesting that minimal performance differences existed between groups.

### 3.3 Anatomical imaging and motion results

A total of 24 pmTBI received a CT scan as part of routine care, with 3/24 pmTBI diagnosed with a positive intracranial finding on CT. One other additional patient was identified as having trauma‐related findings on structural MRI scans (Table S3). There were no significant differences between pmTBI and HC on mean FD (all *p*'*s* > .10) across all three imaging modalities. However, a conservative approach of using FD as a covariate for all analyses was adopted given concerns regarding motion in pediatric imaging data (Power, Schlaggar, & Petersen, 2015).

### 3.4 Multisensory task analyses: Cue phase

**Figure 3:**
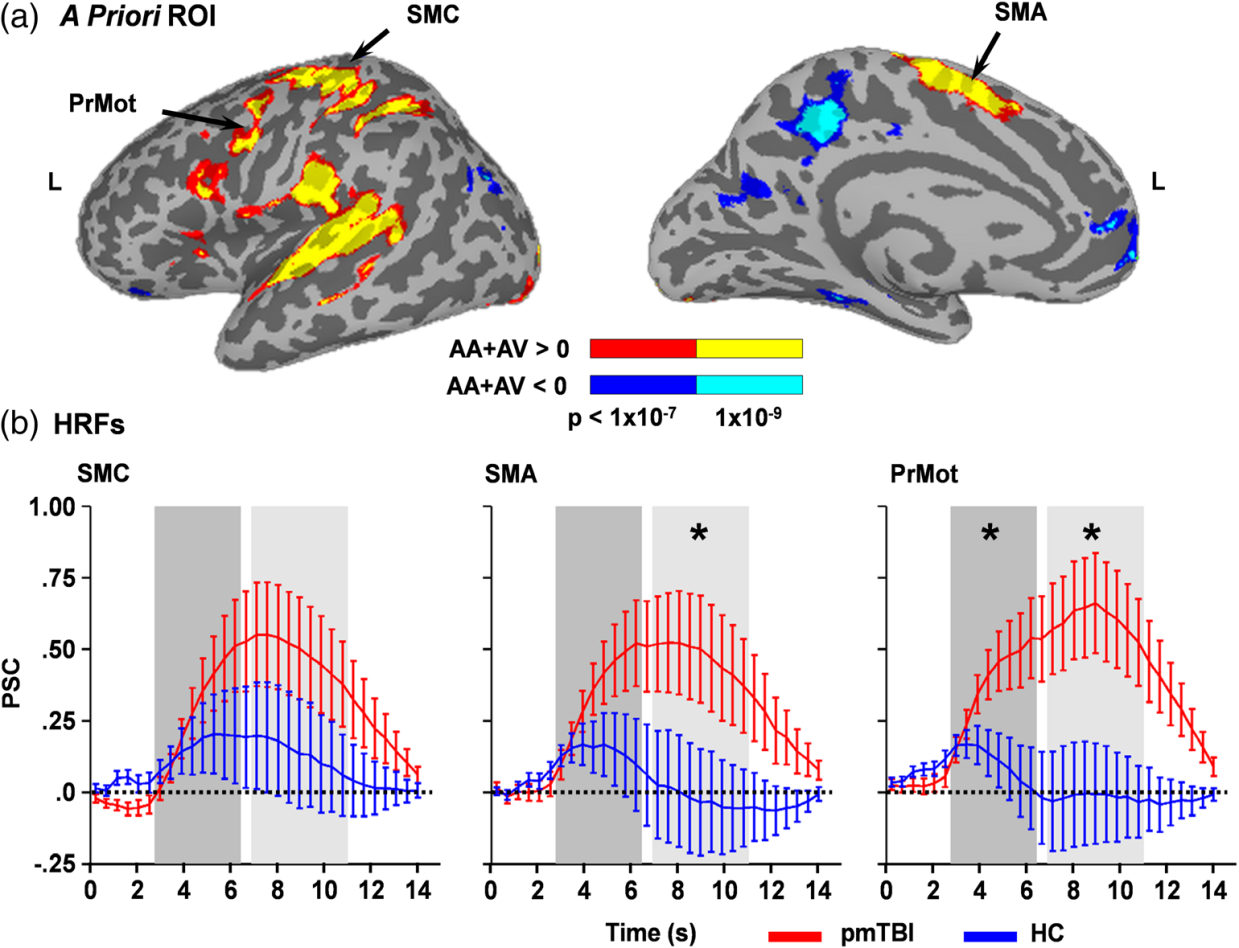
HRFs for regions of interest. Panel a depicts a priori regions of interest (ROI) within motor circuitry including the left sensorimotor cortex (SMC), bilateral supplementary motor area (SMA) and left premotor area (PrMot). ROI were derived from a contrast comparing the active trials of the multisensory task (AA: attend‐auditory; AV: attend‐visual) relative to baseline collapsing across both pediatric mild traumatic brain injury patients (pmTBI) and healthy controls (HC). Inflated views of increased activation relative to baseline are denoted in warm colors (red: *p* < 1 × 10^−7^; yellow: *p* < 1 × 10^−9^) for the lateral and medial portions of the left (L) hemisphere, whereas decreased activation is denoted in cool colors (blue: *p* < 1 × 10^−7^; cyan: *p* < 1 × 10^−9^). Percent signal change (PSC) values for the entire BOLD hemodynamic response function (HRF; b) are presented separately for pmTBI (red line) and HC (blue line) during the cue phase. Shaded bars indicate the peak (dark gray; 2.76–6.44 s) and inhibitory (light gray; 6.90–11.04 s) phases of the HRF, with asterisks denoting significant group differences (pmTBI > HC). Error bars represent the standard error of the mean [Color figure can be viewed at http://wileyonlinelibrary.com]

Our first set of a priori analyses (*N* = 42 pmTBI; *N* = 50 HC) examined predictions of reduced motor inhibition (i.e., increased activity) following pmTBI within the left premotor cortex, bilateral SMA and left SMC during the cue phase (Figure 3a; Figure S2). Results indicated that a priori hypotheses were supported, with pmTBI exhibiting increased activity during the peak phase in the premotor cortex (*F*_1,89_ = 5.70; *p* = .019) relative to HC. During the inhibitory phase, pmTBI again exhibited increased activation relative to HC in both the SMA (*F*_1,89_ = 4.36; *p* = .040) and premotor cortex (*F*_1,89_ = 7.60; *p* = .007). There were no Group × Visit interactions.

**Figure 4:**
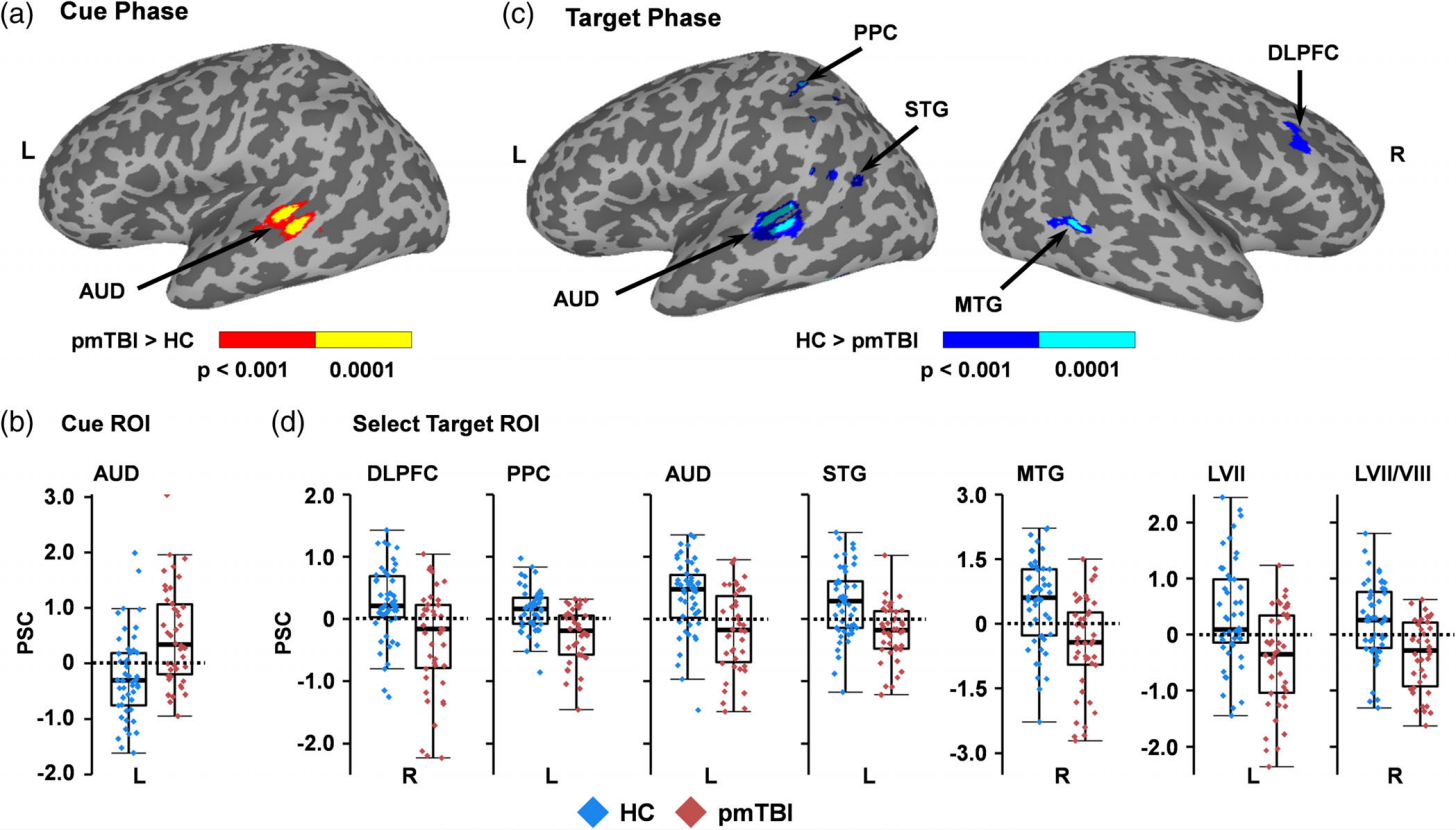
Cue and target phase activation and PSCs. Panel a depicts increased activation (red: *p* < .001; yellow: *p* < .0001) within the left (L) auditory cortex (AUD) for pediatric mild traumatic brain injury patients (pmTBI) relative to healthy controls (HC) during the inhibitory phase of the cue presentation. Panel b displays box and scatter plots of the percent signal change (PSC) for each group in this region (pmTBI: red; HC: blue). In contrast, HC exhibited increased activation (blue = *p* < .001; cyan = *p* < .0001) within the right (R) dorsolateral prefrontal cortex (DLPFC), L posterior parietal cortex (PPC), L AUD, L superior (STG), R middle (MTG) and L inferior (not pictured or presented) temporal gyrus, as well as L Lobule VII (LVII; not pictured) and R Lobule VII/VIII (LVII/VIII; not pictured) of the cerebellum during the target phase (c). Panel d displays box and scatter plots for selected regions [Color figure can be viewed at http://wileyonlinelibrary.com]

Results from 2 × 2 [Group (pmTBI vs. HC) × Visit (Sub‐acute vs. Early chronic)] whole‐brain ANCOVAs were not significant following false positive correction during the peak phase of the cue. A main effect of Group (pmTBI > HC) was observed in the left secondary auditory cortex (BAs 21/22; 1,164 μl) during the inhibitory phase of the cue HRF (Figure 4a,b; Figure S3). No other main effects or interactions were significant.

### 3.5 Multisensory task analyses: Target phase

A 2 × 2 × 2 [Group (pmTBI vs. HC) × Visit (Sub‐acute vs. Early chronic) × Phase (Peak activation vs. Late peak)] whole‐brain ANCOVA indicated a main effect of Group again within left secondary auditory cortex (BAs 21/22; 1,399 μl) during the processing of targets (Figure 4c,d; Figure S4). However, in contrast to cue‐related activity, pmTBI now exhibited reduced activation relative to HC. Similarly, the right dorsolateral prefrontal cortex (DLPFC: BA 9; 586 μl), left posterior parietal cortex (961 μl), left superior temporal gyrus (BA 22; 729 μl), right posterior middle temporal gyrus (BAs 21/22/37; 987 μl) and left inferior temporal/fusiform gyrus (BAs 20/37; 789 μl) also exhibited increased activation for HC relative to pmTBI. This effect was also present in left Lobule VII (582 μl) and right Lobules VII/VIII (1,219 μl) of the cerebellum. No other main effects or interactions were significant during target analyses.

### 3.6 Relationship between neurovascular deficits and clinical outcomes

A binary logistic regression determined whether differences in BOLD response within motor circuitry in the inhibitory phase following cue presentation (weighted mean of SMA and premotor cortex), cue‐related or target‐related activity could classify pmTBI from HC. Results indicated weighted SMA and premotor activity (β = −1.16; Wald = 7.21; odds ratio = 0.32; *p* = .007), cue‐related activity (β = 0.95; Wald = 3.91; odds ratio = 2.57; *p* = .048), and target activity (β = −2.59; Wald = 10.86; odds ratio = .08; *p* = .001) were significant predictors in classifying patients (73.8%) from HC (76.0%) with a total accuracy of 75.0% (Δ = 20.7%) relative to baseline model.

Next, negative binomial (PCS, behavior) or gamma (quality of life) regression models assessed relationships between task‐related BOLD abnormalities and primary outcomes at 4 months in pmTBI only. None of the models were significant when controlling for multiple comparisons (all *p*'s > .10). Post hoc models also examined the relationship between sleep and depression given unpredicted persistence in symptomatology. While the results for depression were negative, findings indicated a significant association between motor circuit activity and clinical measures of sleep at the early chronic phase (Wald = 5.59, IRR = 0.78, *p* = .018), whereas whole brain results from the cue and target phase were not significant.

### 3.7 Functional connectivity analyses

Two 2 × 2 [Group (pmTBI vs. HC) × Visit (Sub‐acute vs. Early chronic)] whole‐brain ANCOVAs were performed on connectivity data using the significant SMA and left premotor cortex ROI as seeds (*N* = 41 pmTBI; *N* = 47 HC). There were no significant interactions or main effects of Group with either analyses.

### 3.8 CBF analyses

There were no significant main effects or interactions for the 2 × 2 [Group (pmTBI vs. HC) × Visit (Sub‐acute vs. Early chronic)] ROI within the motor circuitry (*N* = 41 pmTBI; *N* = 48 HC). Similarly, whole‐brain ANCOVA for CBF was not significant following family‐wise error correction (see Figure S5 for effect sizes).

## 4 DISCUSSION

To date, no objective neurobiological biomarkers exist from which caretakers can predict biological recovery from pmTBI, complicating decisions about return to learn/sports/play (Mayer, Kaushal, et al., 2018). Both animal (Bayly et al., 2006; Huh, Widing, & Raghupathi, 2008) and human (Hessen, Nestvold, & Anderson, 2007) studies have shown that the developing brain may be more susceptible than the developed brain to diffuse injury in more severe trauma. Contrary to a priori predictions, BOLD deficits during a proactive response inhibition task persisted for up to 4 months post‐injury rather than improving. Specifically, motor and sensory cortex were hyperactive during active preparation for inhibition (cue phase), followed by hypoactivation within motor, sensory, and heteromodal prefrontal cortex during the actual processing of multisensory targets. In contrast, resting state abnormalities were not observed within motor circuitry, and deficits in the BOLD response occurred in conjunction with normal CBF.

Clinically, there were no differences across patient groups in terms of demographics, measures of effort or premorbid reading ability. Not surprisingly, pmTBI reported increased PCS sub‐acutely, which resolved, but did not fully return to HC levels, 4 months post‐injury. Of particular note were increased sleep disturbances and depression, both of which are common injury sequelae (Sullivan, Edmed, Allan, Karlsson, & Smith, 2015). Similarly, parental reports of increased PCS and behavioral disturbances remained elevated at 4 months post‐injury, and further included a decreased quality of life. Similar to other sub‐acute studies (Babikian et al., 2011), subtle deficits were observed in the domains of processing speed (nonsignificant trends) and attention, with the latter persisting up to 4 months post‐injury. Collectively, current and previous (Sesma, Slomine, Ding, & McCarthy, 2008; Yeates et al., 2012) clinical results suggest incomplete resolution of symptoms, emotional sequelae, and cognitive deficits after the traditional period of presumed recovery (i.e., a few weeks) in pmTBI.

Individual hemodynamic responses were disambiguated in the current study during proactive inhibition (following cues) and subsequent target processing. Hyperactivation of motor circuitry and sensory cortex was observed following cues, which has previously been interpreted to reflect inhibitory failure (Li et al., 2013; Mayer et al., 2016), a common behavioral symptom of pmTBI (Li & Liu, 2013) with documented neurological correlates such as impaired long‐interval intracortical inhibition (Seeger et al., 2017). Although a relationship was observed between motor hyperactivation and sleep disturbances, this result should be interpreted with caution given their unplanned nature. In contrast, decreased activation was observed in motor and sensory cortex during target processing, similar to previous results observed during auditory attention (Yang et al., 2012) and working memory (Hammeke et al., 2013; Keightley et al., 2014) tasks following pmTBI. Regions of hypoactivation for pmTBI included the DLPFC, a key node for the implementation of cognitive control (Braver, 2012). Hyperactivation during target processing also occurred in conjunction with near normal task performance, suggesting that physiological markers of inhibition may be more sensitive than behavioral performance in this relatively easy task.

Previous studies have indicated a reversal in coupling deficits as a function of recovery (Hammeke et al., 2013) whereas current findings remained statistically unchanged over a 4‐month follow‐up window. Key differences between these two longitudinal studies include injury mechanism, severity of injury, sample size, and point of care (athletic vs. emergency room samples), the latter of which has been shown to influence recovery trajectory for symptom provocation in some settings (Mayer et al., 2018). One potential explanation for current results is impaired cognitive planning in which neuronal activity is not appropriately suppressed in pmTBI following a 100% predictive cue, in conjunction with a failure to release inhibition once targets are presented. Alternatively, pmTBI could result in a delayed inhibitory response (i.e., during targets) or a physiological delays in task‐related coupling of neurovascular activity.

To the latter point, traumatic microvascular impairment is a growing area of interest (Galgano et al., 2015) with preclinical studies reporting a reduction in capillary number/diameter (Park, Bell, Siddiq, & Baker, 2009) and reduced cerebral blood flow (Barkhoudarian et al., 2016) following injury. However, in contrast to previous pmTBI studies in sub‐acute pmTBI, there was no evidence of either hypoperfusion (Agrawal et al., 2005; Maugans et al., 2012) or hyperperfusion (Barlow et al., 2017; Stephens et al., 2018) post‐injury, reducing the likelihood of a pure vascular trauma for explaining current BOLD response abnormalities. Importantly, we cannot rule out trauma‐related changes in cerebral vascular reactivity (Metting et al., 2009) or alterations in neurovascular control using collected data. Specifically, increased expression of glial fibrillary acidic protein has been observed in human and animal models of injury (Budde, Janes, Gold, Turtzo, & Frank, 2011; Okonkwo et al., 2013) and is associated with therapeutic outcome (Kochanek et al., 2018). Astrocytes also serving a critical role in mediating the BOLD response (Figley & Stroman, 2011).

A strength of the current study was the inclusion and equivalent scanning (i.e., both time‐points) of an equally powered, typically developing control cohort. This reduced potential confounds associated with neurodevelopment, as cortical thickness, functional connectivity and cerebral blood flow have all been shown to vary with age (Paus, 2007; Satterthwaite et al., 2014). However, several limitations of the study warrant consideration. Foremost, similar to resting state data, proactive response inhibition tasks provide limited information for quantifying task performance (i.e., false positive rate only). Importantly, only a small minority of individuals from either group made false positives errors across both study visits. More challenging inhibition tasks may have detected additional group differences. Second, a post‐labeling delay of 1,800 ms was selected for our adolescent sample based on a priori predictions of hypoperfusion, slightly longer than recommended values for this age range (Alsop et al., 2015). Third, independent measures of physiological noise were not collected or corrected for in our analyses, and we did not record menstrual cycle. Fourth, we performed connectivity analyses only during resting state data secondary to concerns regarding unmodeled task related activity in residualized task data. Finally, significant individual variation exists in the time‐course of recovery for pmTBI (Kirkwood et al., 2008; Yeates et al., 2009). Although deficits in the BOLD response were able to classify patients from controls with moderate accuracy, the majority of current findings should be construed as pertaining to groups of participants rather than individual subjects.

## 5 CONCLUSION

In summary, head trauma is a particularly relevant public health concern for children and adolescents given overall incidence rates and the potential deleterious effects on neurodevelopment (Rausa et al., 2018). BOLD abnormalities may result from impaired neuronal function, impaired neural control of microvessels, direct damage to the vascular system, metabolic disruptions, changes in cerebral blood flow, or a combination of these factors following trauma (Mayer, Kaushal, et al., 2018). The current study highlights the need for multimodal neuroimaging studies to capture the dynamic and multifaceted nature of injury that has been observed in animal models (Barkhoudarian et al., 2016). Objective deficits in BOLD response may serve as a potential biomarker that persist up to 4 months post‐injury, suggesting a longer physiological recovery from injury in children (Mayer et al., 2012). Future work is needed to ensure that these findings replicate in a larger independent sample, as well as to examine longer follow‐up periods.

## CONFLICT OF INTEREST

The authors report no competing interests.

## Supporting information




**Appendix S1** Supporting Information

## Data Availability

The Impact of Diffuse Mild Brain Injury on Clinical Outcomes in Children. The data that support the findings of this study will be openly available in FITBIR at http://fitbir.nih.gov, reference number FITBIR‐STUDY0000339 at the conclusion of this study.
