# Supplementary material for: Proactive inhibition deficits with normal perfusion after pediatric mild traumatic brain injury
Source: Hum Brain Mapp. 2019 Aug 28;40(18):5370–81. doi: 10.1002/hbm.24778 (PMC6864901; doi:10.1002/hbm.24778)
Supplement: Supplementary file 1 — Appendix S1 Supporting Information [file HBM-40-5370-s001.docx]

**Supplemental Materials**

**Methods**

*Sample Characteristics*

Fifty patients with pediatric mild traumatic brain injury (pmTBI; 12 – 18 years old) and 53 age and sex-matched healthy controls (HC) were enrolled into the study. Two pmTBI failed screenings between assessment periods (pregnancy and repeat concussion) and four participants (pmTBI = 2; HC = 2) were excluded due to data acquisition errors. One pmTBI was unable to follow task instructions and four additional participants (TBI = 3; HC = 1) were identified as motion outliers (greater than 3 times interquartile range) for mean framewise displacement (FD) relative to their cohort. Thus, there were a total of 42 pmTBI (25 males; mean age 15.73±2.14) and 50 HC (31 males; 15.31±1.99) included during task-based analyses.

An additional pmTBI patient and 3 HC were excluded as motion outliers during resting state analyses, leaving 41 pmTBI (24 males; mean age = 15.65±2.11) and 47 HC (28 males; mean age = 15.48±1.93) in this cohort. Finally, 1 pmTBI and 2 HC from the task cohort were excluded as motion outliers during CBF quality assurance procedures, for a final sample of 41 pmTBI (24 males; mean age = 15.67±2.13) and 48 HC (29 males; mean age = 15.39±1.97).

*Clinical and Behavioral Measures*

A battery of clinical and neuropsychological measures was administered to pmTBI and HC at each visit. Measures included previous medical history, the Alcohol, Smoking and Substance Involvement Screening Test (ASSIST; WHO Group, 2002), self and parent reports of concussion symptom severity for both retrospective and current periods (Post-Concussion Symptom Inventory [PCSI]; Gioia, Collins, & Isquith, 2008; Gioia, Schneider, Vaughan, & Isquith, 2009), Patient Reported Outcomes Measurement Information System (PROMIS) for sleep (Buysse et al., 2010), anxiety, and depression (Pilkonis et al., 2011), a brief pain rating (0-10 Likert scale; Farrar, Young, Jr., LaMoreaux, Werth, & Poole, 2001), self-report of Tanner stage of development (Kriz et al., 2016), Headache Impact Test (HIT-6; Kosinski et al., 2003), the Strengths and Difficulties Questionnaire (SDQ; Goodman, 1997), Conflict and Behavioral Questionnaire (CBQ; Prinz, Foster, Kent, & O'Leary, 1979), quality of life (Pediatric Quality of Life Inventory-PedsQL Generic Core; Varni, Seid, & Rode, 1999), and the Glasgow Outcome Scale Extended (GOS-E; Beers et al., 2012), Pediatric Revision. Parental distress was measured with the Brief Symptom Inventory (BSI-18; Derogatis & Fitzpatrick, 2004). The urine screen was sensitive for amphetamines, cocaine, marijuana, methamphetamines, opiates, phencyclidine, benzodiazapines, barbituates, methadone and methylenedioxy-methamphetamine.

The cognitive battery included tests of premorbid cognitive ability (WRAT-4; Wilkinson & Robertson, 2006), a shortened measure of effort (Test of Memory Malingering – TOMMe10; Denning, 2012), the Cogstate battery (Cromer, Schembri, Harel, & Maruff, 2015), and selected tests from the Delis-Kaplan Executive Function System (DKEFS; Delis, Kaplan, & Kramer, 2001) and Wechsler Intelligence Scales depending on initial age at assessment. Specifically, the Wechsler Adult Intelligence Scale – IV (WAIS-IV; Wechsler, 2008) was used for participants 16-18 year old at enrollment whereas the Wechsler Intelligence Scale for Children – V (WISC-V; Wechsler, 2014) was used for participants 12-15 year olds at enrollment. Composite measures of attention (DKEFS color-word interference conditions 1-3), processing speed (WAIS-IV/WISC-V digit symbol coding and symbol search), working memory (WISC-V/WAIS-IV digit span backwards trial) and executive function (DKEFS trail making test condition 4, verbal fluency, color-word interference condition 4) were compiled to create specific cognitive domains. All neuropsychological tests were selected based on whether they were a CDE, whether they contained appropriate normative data for the current age range, and based upon our primary domains of interest.

*MR Imaging Parameters*

All participants underwent imaging on a 3T Siemens Trio scanner with a 32-channel head coil. Foam padding was used to minimize head motion. A high resolution 5-echo Magnetization Prepared Rapid Acquisition Gradient Echo (MPRAGE) T_1_–weighted [repetition time (TR) = 2530 ms; echo times (TE) = 1.64, 3.5, 5.36, 7.22, 9.08 ms; inversion time (TI) = 1200 ms; flip angle = 7°; number of excitations (NEX) = 1; slice thickness = 1 mm; field of view (FOV) = 256 mm; matrix size = 256 x 256; isotropic voxels = 1 mm] was collected in addition to a T_2_–weighted sequence [TR = 15500 ms; TE = 77 ms; flip angle = 155°; NEX = 1; slice thickness = 1.5mm; FOV = 220 mm; matrix size = 192 x 192; voxel size = 1.15 x 1.1.5 x 1.5 mm]. Susceptibility weighted images (SWI) were collected using a single T_2_-weighted gradient echo sequence [TR = 28 ms; TE = 20.0 ms; flip angle = 15; NEX = 1; slice thickness = 1.5 mm; FOV = 192 x 256; matrix size = 192 x 256; 88 interleaved slices; 1.00 x 1.00 x 1.50 mm voxels]. Fluid attenuated inversion recovery (FLAIR) data were collected using the following parameters [TR = 10380 ms; TE = 88.0 ms; TI = 2500 ms; flip angle = 140; NEX = 1; slice thickness = 3 mm; FOV = 256; matrix size = 320 x 320; 50 interleaved slices; 0.80 x 0.80 x 3.00 mm voxels].

Functional data were acquired with a single-shot, gradient-echo echoplanar pulse sequence [TR = 460 ms; TE = 29 ms; flip angle = 44°; multiband acceleration factor = 8; NEX = 1; slice thickness = 3 mm; FOV = 248 mm; matrix size = 82 x 82] over two runs of the task, with fifty-six interleaved 3 mm slices acquired for whole-brain coverage (voxel size: 3.02 x 3.02 x 3.00 mm). A single run of resting state data was also collected. A reference image with multiband acceleration factor set to one (i.e., no acceleration) was also acquired for each run to facilitate registration with native T_1_-weighted anatomical image due to increased grey-white contrast. To account for susceptibility artifacts in the gradient echo data, two spin-echo field mapping sequences [TR = 7220 ms; TE = 73 ms; flip angle = 90°; refocus flip angle = 180°; slice thickness = 3 mm; FOV = 248 mm; matrix size = 82 x 82; 56 interleaved slices; 3.02 x 3.02 x 3.00 mm voxels] with reversed phase encoding directions (A 🡪 P; P 🡪 A) were collected.

Finally, a pseudo-Continuous Arterial Spin Labeling (pCASL; 45 tagged/untagged images) sequence was acquired [TR  = 4250 ms; TE  =  11 ms; label offset = 90 mm; NEX = 1; slice thickness = 5 mm with 20% gap; bandwidth = 2790 Hz/Px; labeling duration = 1665 ms] with 20 interleaved slices for whole brain coverage (voxel size = 3.44 × 3.44 × 6.00 mm]. A proton density sequence was also acquired to estimate T_1_ magnetization and scale CBF on a voxel-wise basis, with the post-labeling delay (PLD) and TR (5200 ms) being the only parameters that varied across the pCASL (PLD = 1800 ms) and PD scans (PLD = 3400 ms).

A one-sample t-test was performed with PSC data from the attend-auditory and attend-visual conditions to define motor circuitry and sensory areas. Clusters were defined at a higher threshold (*p* < 1.0x10^7^) given that the contrast was against baseline state (i.e., visual fixation and gradient noise).

***Supplemental Results***

One pmTBI and no HC failed the measure of effort (TOMMe10 score = 6). However, this participant’s data were retained in further analyses after examination of neuropsychological data revealed that a) their traditional neuropsychological measures (*t*-scores) were mostly > 40 (therefore normal), and b) their scores remained relatively constant, or improved, across sub-acute and early chronic assessments. GLMs on retrospective data (uncorrected) showed significant differences between HC and pmTBI for sleep (*Wald*-χ^2^ = 9.60; *p* = 0.002), pain (*Wald*-χ^2^ = 8.12; *p* = 0.004), headache (*Wald*-χ^2^ = 10.06; *p* = 0.002) and child ratings of behaviour (*Wald*-χ^2^ = 4.11; *p* = 0.043) and quality of life (*Wald*-χ^2^ = 6.54; *p* = 0.011).

For secondary clinical measures, GEEs [Group (pmTBI vs. HC) × Time (Sub-Acute vs. Early Chronic)] were performed with retrospective ratings as covariate when collected. There was a main effect of Group for sleep disturbances (*Wald*-χ^2^ = 7.30; *p* = 0.007) and depression (*Wald*-χ^2^ = 6.41; *p* = 0.011), with pmTBI (sleep IRR = 1.18; depression IRR = 1.72) reporting increased symptoms relative to HC. A Group × Time interaction was observed for anxiety (*Wald*-χ^2^ = 8.25; *p* = 0.004), headache (*Wald*-χ^2^ = 8.32; *p* = 0.004) and pain (*Wald*-χ^2^ = 12.13; *p* < 0.001), with significant differences observed between groups at the sub-acute (pmTBI > HC; anxiety IRR = 3.29; headache IRR = 1.17; pain IRR = 6.68; all *p’s* < 0.001) but not early chronic phases.

**References**

Beers, S. R., Wisniewski, S. R., Garcia-Filion, P., Tian, Y., Hahner, T., Berger, R. P. et al. (2012). Validity of a pediatric version of the Glasgow Outcome Scale-Extended. *J.Neurotrauma, 29*, 1126-1139.

Buysse, D. J., Yu, L., Moul, D. E., Germain, A., Stover, A., Dodds, N. E. et al. (2010). Development and validation of patient-reported outcome measures for sleep disturbance and sleep-related impairments. *Sleep, 33*, 781-792.

Cromer, J. A., Schembri, A. J., Harel, B. T., & Maruff, P. (2015). The nature and rate of cognitive maturation from late childhood to adulthood. *Front Psychology, 6*, 704.

Delis, D. C., Kaplan, E., & Kramer, J. H. (2001). *Delis-Kaplan executive function system (D-KEFS)* Psychological Corporation.

Denning, J. H. (2012). The efficiency and accuracy of the Test of Memory Malingering trial 1, errors on the first 10 items of the test of memory malingering, and five embedded measures in predicting invalid test performance. *Arch.Clin.Neuropsychol., 27*, 417-432.

Derogatis, L. R., & Fitzpatrick, M. (2004). The SCL-90-R, the Brief Symptom Inventory (BSI), and the BSI-18.

Farrar, J. T., Young, J. P., Jr., LaMoreaux, L., Werth, J. L., & Poole, R. M. (2001). Clinical importance of changes in chronic pain intensity measured on an 11-point numerical pain rating scale. *Pain, 94*, 149-158.

Gioia, G. A., Collins, M., & Isquith, P. K. (2008). Improving identification and diagnosis of mild traumatic brain injury with evidence: psychometric support for the acute concussion evaluation. *J.Head Trauma Rehabil., 23*, 230-242.

Gioia, G. A., Schneider, J. C., Vaughan, C. G., & Isquith, P. K. (2009). Which symptom assessments and approaches are uniquely appropriate for paediatric concussion? *Br.J.Sports Med., 43 Suppl 1*, i13-i22.

Goodman, R. (1997). The Strengths and Difficulties Questionnaire: a research note. *J.Child Psychol.Psychiatry, 38*, 581-586.

Kosinski, M., Bayliss, M. S., Bjorner, J. B., Ware, J. E., Jr., Garber, W. H., Batenhorst, A. et al. (2003). A six-item short-form survey for measuring headache impact: the HIT-6. *Qual.Life Res., 12*, 963-974.

Kriz, P. K., Stein, C., Kent, J., Ruggieri, D., Dolan, E., O'Brien, M. et al. (2016). Physical Maturity and Concussion Symptom Duration among Adolescent Ice Hockey Players. *J.Pediatr., 171*, 234-239.

Pilkonis, P. A., Choi, S. W., Reise, S. P., Stover, A. M., Riley, W. T., & Cella, D. (2011). Item banks for measuring emotional distress from the Patient-Reported Outcomes Measurement Information System (PROMIS(R)): depression, anxiety, and anger. *Assessment., 18*, 263-283.

Prinz, R. J., Foster, S., Kent, R. N., & O'Leary, K. D. (1979). Multivariate assessment of conflict in distressed and nondistressed mother-adolescent dyads. *J.Appl.Behav.Anal., 12*, 691-700.

Varni, J. W., Seid, M., & Rode, C. A. (1999). The PedsQL: measurement model for the pediatric quality of life inventory. *Med.Care, 37*, 126-139.

Wechsler, D. (2014). Wechsler intelligence scale for children − Fifth edition (WISC-V): Technical and interpretive manual. Bloomington, MN: Pearson Clinical Assessment.

Wechsler, D. (2008). *Wechsler adult intelligence scale-fourth* San Antonio: Pearson.

WHO Group. (2002). The alcohol, smoking and substance involvement screening test (ASSIST): development, reliability and feasibility. *Addiction, 97*, 1183-1194.

Wilkinson, G. S., & Robertson, G. J. (2006). *WRAT 4: Wide range achievement test; professional manual* Psychological Assessment Resources, Incorporated.

**Supplemental Table 1:** Sample size and demographic information for various imaging modalities.

| Exclusion Reason | pmTBI N (sex, age) | HC N (sex, age) |
| --- | --- | --- |
| Enrolled | 50 (30 M, 15.44±2.14) | 53 (34 M, 15.22±2.00) |
| Failed EC screening | 48 (29 M, 15.44±2.15) | 53 (34 M, 15.22±2.00) |
| Acquisition errors | 46 (28 M, 15.50±2.18) | 51 (32 M, 15.25±2.02) |
| Poor behavioral performance | 45 (27 M, 15.57±2.15) | 51 (32 M, 15.25±2.02) |
| Task: motion outlier | 42 (25 M, 15.73±2.14) | 50 (31 M, 15.31±1.99) |
| fcMRI: motion outlier | 41 (24 M, 15.65±2.11) | 47 (28 M, 15.48±1.93) |
| CBF: motion outlier | 41 (24 M, 15.67±2.13) | 48 (29 M, 15.39±1.97) |

Notes: Total N after exclusion (Number of males [M], mean age ± standard deviation). Exclusions are cumulative through Task motion outliers, with the connectivity (fcMRI) and cerebral blood flow (CBF) sample size derived from those who completed the task. EC = early chronic; HC = healthy control; pmTBI = pediatric mild traumatic brain injury.

**Supplemental Table 2:** Retrospective measures.

|  | Outcome | HC | pmTBI |
| --- | --- | --- | --- |
| PCSI | P | 4(1-10.5) | 5(1-15.25) |
| PCSI (Parent) | P | 2(0-6) | 2(0-6) |
| PROMIS Sleep * | S | 14.04±4.04 | 17.24±5.94 |
| PROMIS Anxiety | S | 3(1-6) | 2.5(0-6.75) |
| PROMIS Depression | S | 1(0-3.75) | 1(0-5.5) |
| Pain Scale * | S | 0(0-1) | 0.5(0-3) |
| HIT-6 * | S | 44(38-50) | 50(42-55) |
| CBQ * | P | 0(0-2) | 1(0-3) |
| CBQ (Parent) | P | 1(0-3) | 1(1-4) |
| SDQ (Parent) | S | 5(2-8) | 5(3-8.5) |
| PedsQL * | P | 87.5±9.04 | 82.27±10.4 |
| PedsQL (Parent) | P | 85.45±12.21 | 79.74±15.39 |

Notes: CBQ = Children’s Behavior Questionnaire; HC = healthy control; HIT-6 = Headache Impact Test; P = primary; PCSI = Post-Concussion Symptom Inventory; PedsQL = Pediatric Quality of Life Inventory; pmTBI = pediatric mild traumatic brain injury; PROMIS = Patient-Reported Outcomes Measurement Information System; S = secondary; SDQ = Strengths and Difficulties Questionnaire. Data are either formatted at mean ± standard deviation or median (interquartile range). Asterisks indicate significance.

**Supplemental Table 3:** Summary of traumatic CT/MRI Findings

| Age | Sex | Injury Mechanism | CT Finding | MRI Finding |
| --- | --- | --- | --- | --- |
| 15 | M | Fall during basketball practice | NA | Right parietal bone fracture; right parietal subdural hematoma (1, 10 x 15 x 20 mm); right parietal subarachnoid hemorrhage (18 x 15 x 9 mm); probably minimal edema surrounding the contusion. |
| 13 | M | Fall while skateboarding | CT notes unavailable, but referral indicated: ‘CT […] showed left temporal skull fracture.’ | Left mastoid temporal bone fracture; 1, left parietal, small (27x6 mm) subdural hematoma; high right frontal white matter contusion (6mm). |
| 16 | F | Motor vehicle accident | Extra-axial fluid collections over the lateral/temporal cerebral convexities bilaterally, measuring up to 3 mm on the right and 2 mm on the left. No midline shift. Basilar cisterns are patent. No hydrocephalus. Incidentally noted cavum septum et vergae. No large acute ischemic infarction is identified. Dural venous sinuses appear normal. Intraorbital contents are normal. Left frontal scalp, left temporal scalp, and left cheek soft tissue contusions. No acute osseous abnormality. Paranasal sinuses and mastoid air cells are clear. | 2, left frontal and temporal subcortical white matter hemorrhagic traumatic axonal injury, 1-2 cm longitudinal. |
| 14 | M | Fall during bull riding | Intraparenchymal hemorrhage is noted in the white matter of the frontal lobe superior frontal gyrus near the vertex with surrounding mild edema is measured 1.7 x 0.7 x 2.0 cm (AP by transaxial by cc). Trace hyperdensity favored to represent subarachnoid hemorrhage is noted near the vertex on image #142/169. Punctate hemorrhage is noted in the right anterior insula. No hydrocephalus or herniation. | 2, anterior right frontal subcortical and high right insular cortex contusions (20x10mm and 8x6mm); 5, left insular cortex and right medial temporal cortex and along left fornix, and bilateral midbrain, punctate lesions mostly oriented in AP linear fashion; Some of the lesions described as DAI could be grey matter micro-bleeds. |

Notes: F = female; M = male; NA = not applicable.


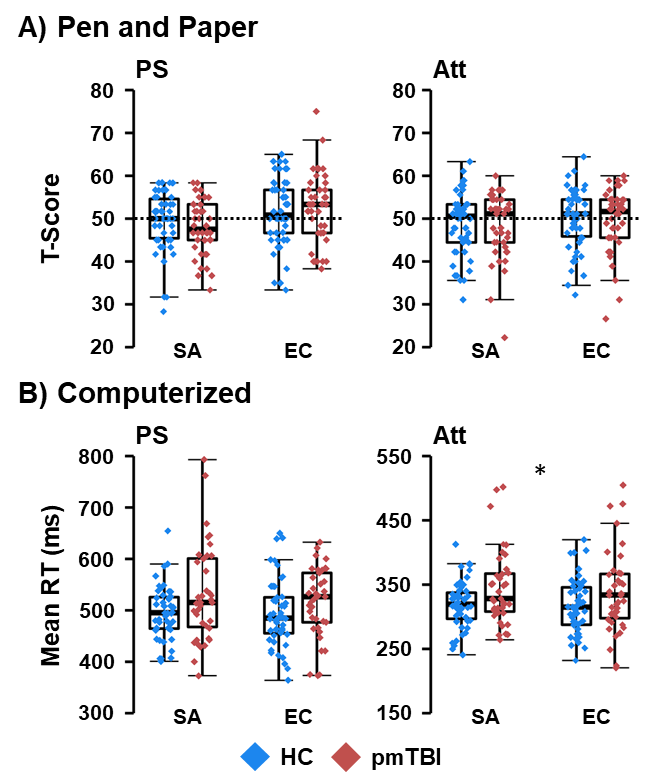


**Supplemental Figure 1:** Scatter box plots for pen and paper (Panel A) and computerized (Panel B) measures of processing speed (PS) and attention (Att) for healthy control (HC) and pediatric mild traumatic brain injury patients (pmTBI) at sub-acute (SA) and early chronic (EC) visits. Significant main effects of group are denoted with an asterisk (*).

**Supplemental Figure 2:** Panel A depicts *a priori* regions of interest (ROI) within motor circuitry including the left sensorimotor cortex (SMC), bilateral supplementary motor area (SMA) and left premotor area (PrMot). ROI were derived from a contrast comparing the active trials of the multisensory task (AA: attend-auditory; AV: attend-visual) relative to baseline collapsing across both pediatric mild traumatic brain injury patients (pmTBI) and healthy controls (HC). Inflated views of increased activation relative to baseline are denoted in warm colors (red: *p* < 1×10^-7^; yellow: *p* < 1×10^-9^) for the lateral and medial portions of the left (L) hemisphere, whereas decreased activation is denoted in cool colors (blue: *p* < 1×10^-7^; cyan: *p* < 1×10^-9^). Percent signal change (PSC) values for the entire hemodynamic response function (HRF; Panel B) are presented separately for pmTBI (red lines) and HC (blue lines) during the cue phase. As opposed to Figure 3, HRFs are plotted separately for sub-acute (SA; sharp colors) and early chronic (EC; muted colors) time points for each group. Shaded bars indicate the peak (dark grey) and inhibitory (light grey) phases of the HRF, with asterisks denoting significant group differences (pmTBI > HC). Error bars represent the standard error of the mean.

**Supplemental Figure 3:** Panel A depicts increased activation (red: *p* < 0.001; yellow: *p* < 0.0001) within the left (L) auditory cortex (AUD) for pediatric mild traumatic brain injury patients (pmTBI) relative to healthy controls (HC) during the inhibitory phase of the cue presentation. Panel B displays box and scatter plots of the percent signal change (PSC) for this region in each group (pmTBI: red; HC: blue). As opposed to Figure 4B, PSCs are plotted separately for sub-acute (SA) and early chronic (EC) time points for each group.

**Supplemental Figure 4:** Panel A depicts increased activation (blue = *p* < 0.001; cyan = *p* < 0.0001) within the right (R) dorsolateral prefrontal cortex (DLPFC), left (L) posterior parietal cortex, L auditory cortex (AUD), L superior (STG) R middle (MTG) and L inferior (ITG; not pictured) temporal gyrus, and L Lobule VII (LVII; not pictured) and R Lobule VII/VIII (LVII/VIII; not pictured) of the cerebellum for healthy controls (HC) relative to pediatric mild traumatic brain injury patients (pmTBI) during the target phase of the task. Panel B displays box and scatter plots of the percent signal change (PSC) for each region and group (pmTBI: red; HC: blue). As opposed to Figure 4D, PSCs are plotted separately for sub-acute (SA) and early chronic (EC) time points for each group.

**Supplemental Figure 5:** This figure depicts Cohen’s *d* effect size maps of cerebral blood flow (CBF) on inflated views of the right (R) and left (L) hemispheres for pediatric mild traumatic brain injury (pmTBI) patients and healthy controls (HC) based on data from sub-acute (SA) and early chronic (EC) time points. Effect sizes appear in warm colors (pmTBI > HC) or cool colors (HC > pmTBI). Small effect sizes (*d* = -0.29 to 0.29) are not displayed. For all maps, Cohen’s *d* is adjusted for mean FD. Panel A depicts effect sizes for the main effect (M.E.) of Group whereas Panel B depicts effect sizes for the Group × Time interaction.
